# The role of African buffalos (*syncerus caffer*) in the maintenance of foot-and-mouth disease in Uganda

**DOI:** 10.1186/1746-6148-6-54

**Published:** 2010-12-11

**Authors:** Chrisostom Ayebazibwe, Frank N Mwiine, Kirsten Tjørnehøj, Sheila N Balinda, Vincent B Muwanika, Anna R Ademun Okurut, Graham J Belsham, Preben Normann, Hans R Siegismund, Soren Alexandersen

**Affiliations:** 1 Ministry of Agriculture, Animal Industry and Fisheries, P.O. Box 513, Entebbe, Uganda; 2 Makerere University Institute of Environment and Natural Resources, P.O. Box 7298, Kampala, Uganda; 3 National Veterinary Institute, Technical University of Denmark, Lindholm, DK-4771, Kalvehave, Denmark; 4 Department of Biology, University of Copenhagen, Ole Maaløes Vej 5, DK-2200 Copenhagen N, Denmark; 5 Department of Veterinary Medicine, Faculty of Veterinary Medicine, Makerere University, Box 7062, Kampala, Uganda; 6 National Centre for Foreign Animal Diseases, 1015 Arlington Street, Winnipeg MB R3E 3M4, Canada

## Abstract

**Background:**

To study the role of African buffalos (*Syncerus caffer*) in the maintenance of foot-and-mouth disease in Uganda, serum samples were collected from 207 African buffalos, 21 impalas (*Aepyceros melampus*), 1 giraffe (*Giraffa camelopardalis*), 1 common eland (*Taurotragus oryx*), 7 hartebeests (*Alcelaphus buselaphus*) and 5 waterbucks (*Kobus ellipsiprymnus*) from four major National Parks in Uganda between 2005 and 2008. Serum samples were screened to detect antibodies against foot-and-mouth disease virus (FMDV) non-structural proteins (NSP) using the Ceditest^® ^FMDV NS ELISA. Solid Phase Blocking ELISAs (SPBE) were used to determine the serotype-specificity of antibodies against the seven serotypes of FMDV among the positive samples. Virus isolation and sequencing were undertaken to identify circulating viruses and determine relatedness between them.

**Results:**

Among the buffalo samples tested, 85% (95% CI = 80-90%) were positive for antibodies against FMDV non-structural proteins while one hartebeest sample out of seven (14.3%; 95% CI = -11.6-40.2%) was the only positive from 35 other wildlife samples from a variety of different species. In the buffalo, high serotype-specific antibody titres (≥ 80) were found against serotypes O (7/27 samples), SAT 1 (23/29 samples), SAT 2 (18/32 samples) and SAT 3 (16/30 samples). Among the samples titrated for antibodies against the four serotypes O, SAT 1, SAT 2 and SAT 3, 17/22 (77%; CI = 59.4-94.6%) had high titres against at least two serotypes.

FMDV isolates of serotypes SAT 1 (1 sample) and SAT 2 (2 samples) were obtained from buffalo probang samples collected in Queen Elizabeth National Park (QENP) in 2007. Sequence analysis and comparison of VP1 coding sequences showed that the SAT 1 isolate belonged to topotype IV while the SAT 2 isolates belonged to different lineages within the East African topotype X.

**Conclusions:**

Consistent detection of high antibody titres in buffalos supports the view that African buffalos play an important role in the maintenance of FMDV infection within National Parks in Uganda. Both SAT 1 and SAT 2 viruses were isolated, and serological data indicate that it is also likely that FMDV serotypes O and SAT 3 may be present in the buffalo population. Detailed studies should be undertaken to define further the role of wildlife in the epidemiology of FMDV in East Africa.

## Background

Foot-and-mouth disease (FMD) is a highly contagious viral disease that affects all cloven-hoofed wild and domestic animals [1] and has serious socio-economic consequences [2]. The epidemiology of FMD in Africa is unique, complex and poorly understood. Seven FMDV serotypes have been defined: O, A, C, Asia 1, and the Southern African Territories (SAT) 1, SAT 2 and SAT 3, of which all but Asia 1 have occurred in most East African countries including Uganda [3]. Wildlife hosts, especially African buffalos (*Syncerus caffer*), are believed to play an important role as reservoirs for the SAT serotypes of FMDV [4] and the disease is sometimes transmitted between and within different livestock and wildlife species [5-9].

In Africa, the epidemiology of FMD is complicated by the widespread movement of animals, the wide host range of the virus involving wild and domestic animal reservoirs and the presence of multiple strains and sub-strains. Moreover, the spread of the disease is facilitated by the ability of the virus to survive for relatively long periods in raw meat, raw milk or outside the host [1,10,11]. Infection of cloven-hoofed animals can result in development of a carrier state in which case FMDV may be found in such animals for more than 28 days after infection [12-14], and thus may influence the epidemiology of the disease and interfere with its diagnosis and control. The duration of the carrier state can be prolonged after recovery from acute disease; in the case of cattle for up to 3.5 years [14]. The epidemiology of FMD in wildlife populations has not been fully documented but it has been established that African buffalo herds can harbour the infection for up to 24 years [15]. They act as long term maintenance hosts for the SAT serotypes (SAT 1, SAT 2 and SAT 3) of FMDV with no obvious clinical disease [4,16]. Other cloven-hoofed wildlife species may develop antibodies against FMD infections; however, their roles in excretion, transmission and persistence of FMDV either have not been conclusively studied or have been shown to be less important than the role of the buffalos [7,17,18]. In South Africa, the impala (*Aepyceros melampus*) has been shown to play a potentially significant role in the propagation of FMD outbreaks between livestock and wildlife [19].

FMD outbreaks are often encountered in cattle in Uganda but the roles of different wild and domestic hosts in the maintenance and spread of FMDV have not been exhaustively studied. Available data on seventy-three Ugandan FMD outbreaks, mainly in cattle, and a few isolates from apparently healthy buffalos, indicate that between the years 1958 and 2000, approximately 31% were attributed to serotype O, 26% to A, 25% to SAT 2, 14% to SAT 1, 3% to C and 1% to SAT 3 [3]. FMDV serotypes SAT 1, SAT 2 and SAT 3 have been found in many other sub-Saharan African countries, however, the viruses found in East Africa seem to belong to distinct lineages [20-22]. The possible role played by the African buffalos in the epidemiology of FMDV serotypes other than SATs has not been established, since only one single study in Queen Elizabeth National Park has reported antibodies against serotypes O and A [23], thus further research is required in this field.

This study was undertaken to evaluate the role of African buffalos and other wildlife species in the maintenance of different FMDV serotypes under natural conditions in selected National Parks in Uganda.

## Results

### Antibodies elicited against FMDV NSP

Between 2005 and 2008, 207 samples were collected from African buffalos and 35 samples were collected from other wildlife species (21 impala (*Aepyceros melampus*), 1 giraffe (*Giraffa camelopardalis*), 1 common eland (*Taurotragus oryx*), 7 hartebeest (*Alcelaphus buselaphus*) and 5 waterbuck, (*Kobus ellipsiprymnus*)) in Queen Elizabeth National Park (QENP), Lake Mburo National Park (LMNP), Kidepo Valley National Park (KVNP) and Murchison Falls National Park (MFNP). One hundred and seventy-six out of 207 buffalo samples (85%; 95% CI = 80-90%) tested positive for antibodies against FMDV NSP (Table 1), while only one of seven hartebeest samples (14.3%; 95% CI = -11.6-40.2%) from among those of other wildlife species tested positive in the NSP ELISA.

**Table 1:** Screening of serum samples from wildlife collected in four Ugandan National Parks during 2005-2008 for antibodies against the non-structural proteins of foot-and-mouth disease virus

National Park	Species	Total samples collected	Number of samples tested	Number of positive samples
**MFNP**	Buffalo	53	53	**51 (96%)**
	Waterbuck	5	5	**0 (0%)**
	Hartebeest	7	7	**1 (14%)**
	Giraffe	1	1	**0 (0%)**
**LMNP**	Buffalo	25	19	**18 (95%)**
	Impala	21	21	**0 (0%)**
	Eland	1	1	**0 (0%)**
**KVNP**	Buffalo	42	42	**26 (62%)**
**QENP**	Buffalo	94	93	**81 (87%)**

	Total buffalo	214	207	**176 (85%)**
	Total other species	35	35	**1 (3%)**

**Total**		**249**	**242**	**177**

(MFNP-Murchison Falls National Park, LMNP-Lake Mburo National Park, KVNP-Kidepo Valley National Park, QENP-Queen Elizabeth National Park).

### Screening for serotype-specific antibodies using the Solid Phase Blocking ELISA (SPBE)

Ninety-six percent (131/137) of the buffalo samples tested were apparently positive for antibodies against more than one serotype in the screening dilution 1:5 in SPBE. The proportion of positive samples was higher for serotypes SAT 1, SAT 2, SAT 3 and to a lesser extent serotype O, than for serotypes A, C and Asia 1. One hartebeest tested positive for SAT 1, SAT 2, and SAT 3 (data not shown). Cross reactivity between the different serotypes is known to occur in such assays [17].

### Titration of selected samples in relevant serotype-specific SPBEs

Samples from QENP, MFNP and LMNP were selected for titration on the basis of positive screening results and sufficient volumes with the objective of comparison of results across multiple years. A total of 37 buffalo samples were titrated in the relevant serotype-specific SPBEs as follows; O (27), SAT 1 (29), SAT 2 (32) and SAT 3 (30) as shown in Table 2. In this study, samples with titres of ≥ 80 were considered positive based on the highest dilution at which non-specific reactions tended to disappear and the results of a previous study [24]. All the sera titrated for antibodies against serotypes A, C and Asia 1 had titres below 40 and were therefore considered negative (data not shown), while titres of 80 and above were found in the majority of sera titrated for antibodies against serotypes O (26%; 95% CI = 9.5-42.6%), SAT 1 (79%; 95% CI = 64.6-94.1%), SAT 2 (56%; 95% CI = 39.1-73.4%) and SAT 3 (53%; 95% CI = 35.45-71.2%). The samples positive for antibodies against FMDV serotype O were also positive for at least two of the SAT serotypes. Six of 22 (27%; 95% CI = 8.7-45.9%) samples titrated for antibodies against all three SAT serotypes as well as against serotype O were positive for all four serotypes, while 17 (77%; 95% CI = 59.5-94.6%) were positive for at least two serotypes. Nine of the 24 samples titrated for antibodies against all three SAT serotypes were positive for antibodies against all 3 serotypes, including at least one buffalo in each of QENP, LMNP and MFNP.

**Table 2:** Titres of serotype-specific antibodies against foot-and-mouth disease virus in serum samples from African buffalos collected in three National Parks in Uganda during 2005-2008

National Park	Sample ID	Date	O	SAT 1	SAT 2	SAT 3
**LMNP**	BUF 3	JAN.06	-	-	20	-
	BUF 2	JAN.06	10	20	**80**	-
	BUF 7	JAN.06	-	-	**640**	10
	BUF 1	JAN.07	20	**320**	20	-
	BUF 6	JAN.07	20	40	20	5
	BUF 10	APR.07	**160**	**640**	**80**	**640**
	BUF 9	APR.07	40	**80**	**80**	40
	BUF 11	APR.07	10	20	40	40
	BUF 12	APR.07	-	**160**	-	5
	BUF 6	APR.07	-	**-**	40	-
	BUF 1	OCT.08	-	**640**	-	**80**
	BUF 4	OCT.08	-	**80**	40	20
	BUF 5	OCT.08	-	**80**	-	-
	BUF 6	OCT.08	-	**80**	20	20
**MFNP**	BUF 2	OCT.05	**160**	**320**	**80**	**160**
	BUF 7	OCT.05	5	**320**	**160**	**320**
	BUF 15	OCT.05	**320**	**640**	**160**	**160**
	BUF 2	NOV.06	5	10	**320**	20
	BUF 3	NOV.06	20	**80**	40	**80**
	BUF 7	NOV.06	40	**80**	10	**80**
	BUF 12	OCT.07	40	**80**	**160**	**160**
	BUF 5	OCT.07	20	20	20	**160**
	BUF 20	OCT.07	40	**640**	40	**320**
	BUF 18	OCT.07	**640**	**640**	**320**	**320**
**QENP**	BUF 17	JAN.07	5	**160**	**80**	20
	BUF 37	APR.07	5	20	40	20
	BUF 35	APR.07	-	-	**320**	40
	BUF 8	JUL.07	**80**	**320**	**160**	40
	BUF 9	AUG.07	20	**320**	**320**	**160**
	BUF 3	AUG.07	**160**	**640**	**80**	**320**
	BUF 13	AUG.07	**80**	**640**	**80**	**160**
	BUF 1	OCT.08	5	-	40	**160**
	BUF 2	OCT.08	40	**640**	40	**80**
	BUF 3	OCT.08	10	**80**	**320**	20
	BUF 5	OCT.08	-	-	**80**	40
	BUF 6	OCT.08	10	-	**-**	-
	BUF 9	OCT.08	40	-	**-**	-

**Total**			**7/27 (26%)**	**23/29 (79%)**	**18/32 (56%)**	**16/30 (53%)**

Minus signs (-): results of samples with titres < 5 in the screening test and thus not titrated.

Bold figures: results of samples tested positive (ODP ≥ 80)

### Isolation and identification of FMDV

**Figure 1:**
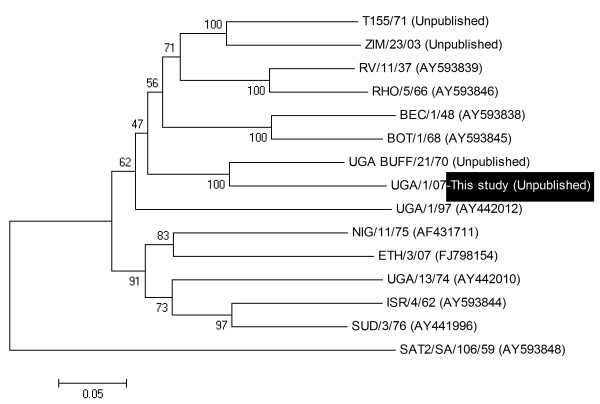
**Neighbour-joining tree depicting VP1 coding sequence relationships of the recent Ugandan SAT 1 isolate (SAT 1/UGA/07) with other SAT 1 reference prototypes from WRLFMD, Pirbright**. Bootstrap values ≥ 50, based on 1,000 replicates are indicated next to the relevant node.

**Figure 2:**
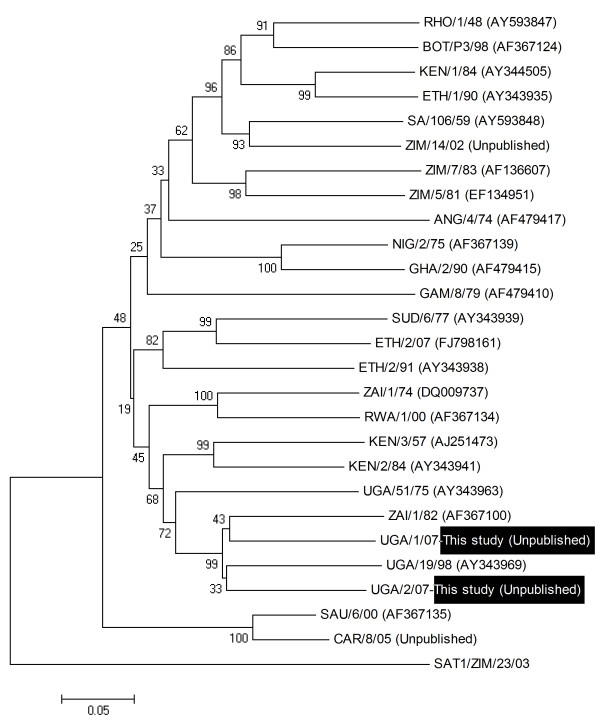
**Neighbour-joining tree depicting VP1 coding sequence relationships of the recent Ugandan SAT 2 isolates (SAT 2/UGA/1/07 and SAT 2/UGA/2/07) with other SAT 2 reference prototypes from WRLFMD, Pirbright**. Bootstrap values ≥ 50, based on 1,000 replicates are indicated next to the relevant node.

**Figure 3:**
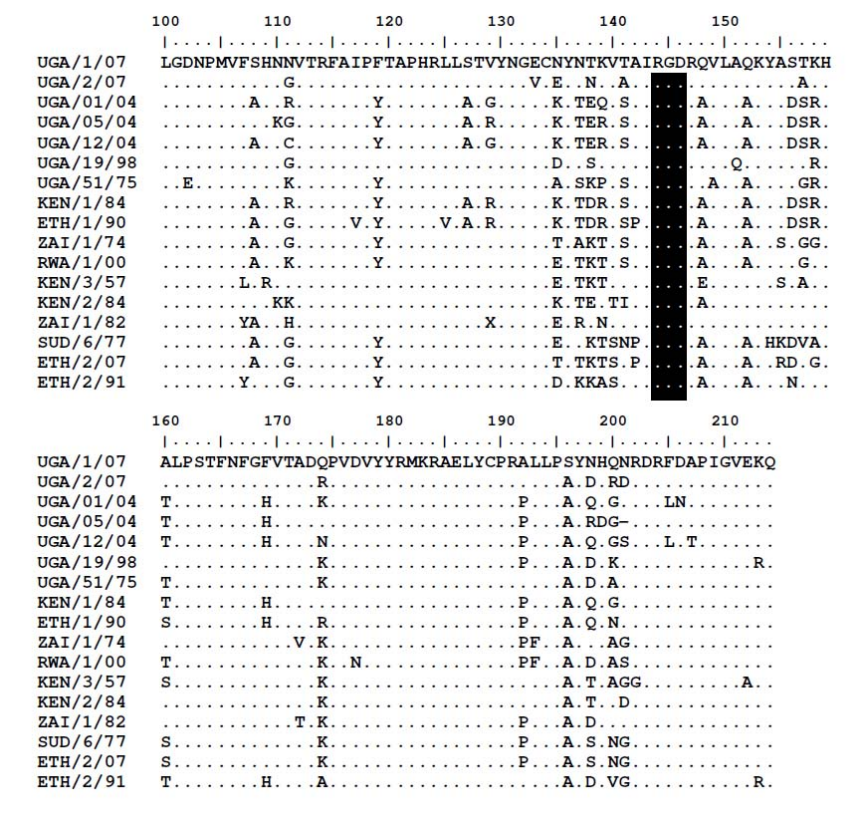
**An alignment of the seventeen deduced amino acid sequences of the C-terminal region of VP1 from the East African SAT 2 FMD reference prototype virus strains and those collected from livestock and African buffalos in Uganda, between the years 2004 and 2007**. Dots indicate sequence identity with master sequence, UGA/1/07 while the "X" in the ZAI/1/82 sequence denotes an ambiguity. The highly conserved 'RGD' cell attachment motifs are indicated by the shaded text box at positions 144-146. The recent buffalo sequences (UGA/1/07) and (UGA/2/07) have a number of amino acid differences from the other SAT 2 sequences including those from cattle in Uganda. These are clustered particularly within the regions 135-160 (G-H loop) and near the extreme C-terminus (residues 190-205). Such differences may be important in influencing the antigenicity of these various strains.

Three FMDV isolates were obtained in primary bovine thyroid cells from among nine buffalo probang samples collected on the same day in January 2007 in QENP, and were identified by antigen ELISA as SAT 1 (1 sample from BUF 17) and SAT 2 (from BUF 6 and BUF 10). BUF 17 had a higher titre of antibodies against SAT 1 (160) compared to those against SAT 2 (80) and SAT 3 (20) (Table 2), while the sera of BUF 6 and BUF 10 were not titrated in the SPBE. Following RT-PCR, the near complete genome sequences were obtained and blasted in the GenBank data base. The sequencing data was entirely consistent with the antigen ELISA results in terms of serotype identification. Due to the limited number of full length SAT serotype sequences that are available, comparative analysis of the virus sequences was restricted to the VP1 coding region. These sequences were compared to reference strains for the defined topotypes [25] to assess the phylogenetic relationships (Figure 1 and 2). The SAT 1 isolate (SAT 1/UGA/1/07, [GenBank HM067706]) was most closely related (pair wise identity of 83%) to a previous isolate obtained from a buffalo in Uganda in 1970 (SAT 1/UGA BUFF/21/70, Knowles et al., unpublished) belonging to the East African topotype IV (Figure 1). The two SAT 2 isolates were closely related to each other (pair wise identity of 90.4%) and grouped with representatives of the topotype X viruses (Figure 2). One of the isolates, SAT 2/UGA/1/07 [GenBank HM067705], was also related to an isolate from cattle in the neighbouring country of Democratic Republic of Congo (pair wise identity 89.5%), while the other, SAT 2/UGA/2/07 [GenBank HM067704], was related to a previous isolate from a buffalo in Uganda (SAT 2/UGA/1998, accession number AY343969) with pair wise identity of 89.6%. There were multiple amino acid differences between the SAT 2 viruses within the G-H loop (residues 140-160) and the C-terminal region of VP1 which correspond to known antigenic sites (Figure 3). The recent SAT 2 buffalo isolates had some amino acid differences, within the hyper-variable regions surrounding the conserved RGD cell attachment motifs, compared to those obtained from post-outbreak slaughtered cattle in Uganda in 2004 [26].

## Discussion

Antibodies against FMDV were detected by both the Ceditest^® ^FMDV NS kit and the SPBE in over 80% of screened buffalo samples. Among the samples of wildlife species other than the buffalos, it was only one from a hartebeest that had detectable antibodies against FMDV. Due to small sample sizes in other tested wildlife species, it is, at this stage, not possible to explain or conclude anything about the importance of these other species relative to buffalos. However, the findings of this study do relate to those of other studies done elsewhere. It has been indicated that a number of wild ruminants become persistently infected with FMDV but it is only the African buffalos that have been shown to spread the infection during the carrier state [16,27]. The situation seems to be different within the impala population in the Kruger National Park in South Africa, where clinical FMD has been reported, and subclinical infections have been shown to occur much more regularly than previously suspected [19]. It is hypothesized that during the acute state of the disease some species may act as intermediaries in the transmission of FMD, mainly between buffalos and cattle [6,18,19]. The current findings concur with reports of very low seroprevalence of antibodies against FMDV in non-buffalo wildlife species (4.4%) compared to buffalos (67.7%) in Eastern Africa [17]. The Ceditest^® ^NSP ELISA seemed to work well in detecting antibodies against FMDV in buffalo samples, with estimates of sensitivity and specificity at 87.7% and 87.3%, respectively [17].

In this study, the majority of the buffalos were positive for antibodies against FMDV NSP during each of the sampling trips between the years 2005 and 2008. This indicates that infection is almost always present in the sampled National Parks. Persistent infections within buffalo herds have been reported to occur in Southern Africa due to most calves becoming infected with the three SAT serotypes, when maternal antibodies wane at 2-6 months of age, thereby creating an opportunity for transmitting the infection to other susceptible species [28-30]. The current findings justify the need to conduct much more in-depth age-stratified longitudinal studies to confirm the serotypes and patterns of FMD in different localities in Uganda.

SPBE screening results (dilution 1:5) were difficult to interpret due to the large percentage (96%) of animals apparently testing positive for antibodies against more than one serotype. However, titrations showed that reactions in the serotype A, C and Asia 1 antibody ELISAs were most likely cross-reactions. This fits well with the lack of any reports of such serotypes in wildlife in Uganda, the almost complete disappearance of serotype C from the world and the fact that serotype Asia 1 has never been reported anywhere on the African continent [3].

This is the first time the SPBEs have been used in an unvaccinated animal population like the buffalos, which probably harbour persistent infections with multiple serotypes. For future studies in endemic conditions, sera should be screened in dilution 1:10, and the SPBE ELISAs should be improved by using more purified antigens and more recent FMDV strains representing the FMDV topotypes currently circulating in Uganda for the production of reagents and positive sera, thereby possibly enhancing the specificity.

Screening of samples by serotype-specific SPBE worked well for selection for further titration, thereby significantly reducing the associated working time and expense. Titrations demonstrated the highest antibody titres against serotypes SAT 1, SAT 2 and SAT 3 with the exception of one out of four buffalos sampled in MFNP in 2007 that had equally high titres against serotypes O and SAT 1.

It is thus evident from the present study, that buffalos were exposed to the FMDV SAT serotypes, and in MFNP probably also to serotype O. These findings suggest that African buffalos may play an important role as natural reservoirs of the SAT serotypes of FMDV in East Africa and are consistent with what has been established in Southern Africa [31-33]. Detection of antibodies against serotype O in this study confirms previous reports of antibodies against other FMDV serotypes than the SATs in buffalos in QENP [23].

The distribution of serotypes varied between the National Parks and between sampling trips. In this study, a large proportion of the buffalo samples had high antibody titres against more than one serotype of FMDV (77%), and this is consistent with previous research findings [17,23,24]. The relative antibody prevalences found in this study (SAT 1 > SAT 2 > SAT 3 > O) differ from those of Bronsvoort et al. [17], who found that antibodies against SAT 2 were the most prevalent, followed by SAT 1 and finally SAT 3, in African buffalos in Eastern Africa. This is likely due to spatial and temporal differences in the distribution of the infection.

Three FMDV isolates consisting of one SAT 1 from a buffalo in one herd and two SAT 2 from buffalos in another herd were obtained from three out of nine African buffalo probang samples collected on the same day in 2007 in QENP indicating the presence of either current or persistent infection. The three isolates were characterised using antigen ELISA and by full-length sequencing. The VP1 coding regions of the two SAT 2 isolates showed that these viruses belonged to the same topotype (X) but different lineages, with 90.4% pair wise identity. One of the SAT 2 isolates (SAT 2/UGA/1/07) was most closely related with a previous isolate (SAT 2/ZAI/1/82 [AF367100]) from cattle in the neighbouring country of Democratic Republic of Congo (89.5% pair wise identity) indicating a possibility of cross-border and wildlife-livestock transmission. The SAT 1 sequence was closest to a representative of the topotype IV isolate obtained in 1970 from a buffalo in Uganda (SAT 1/UGA BUFF/21/70, N. Knowles, unpublished) with a pair wise identity of 83%. It is clear from this study that the viruses obtained are different from each other. These differences may be of particular significance during selection of strains that may be considered for vaccine manufacture and effective control of foot-and-mouth disease due to a range of viruses that may be shared between wildlife and livestock. The isolation and characterization of these viruses from buffalo confirms the presence of SAT 1 and SAT 2 types of FMDV as demonstrated serologically by SPBEs. More molecular epidemiological studies are necessary for precise elucidation of the diversity of FMDV genotypes and the possible challenges involved in matching such strains with those included in vaccines produced for use in Uganda. Molecular studies including the current SAT 1 virus in this study suggest that a unique group of SAT 1 viruses exist in Uganda and, may necessitate a regional approach for effective control [34].

Consistent evidence of antibodies against multiple serotypes of FMDV in several Ugandan National Parks and the isolation of SAT 1 and SAT 2 in QENP in 30% of nine apparently healthy buffalos indicates that wildlife maintains FMDV infections, and thus re-affirms recent findings in buffalo sera collected during 2001-2003 [34]. These findings combined with serological evidence of exposure of cattle grazing in QENP to the SAT serotypes [35] emphasizes the need to study FMDV isolates from these two populations to establish whether FMDV is transferred between them and at which rate.

FMDV serotype SAT 3 was isolated from a buffalo in QENP in 1970 [36] and this study indicates that this serotype may still be present. It is not clear why outbreaks caused by serotype SAT 3 have never been confirmed in cattle, while outbreaks of FMDV SAT 1 and SAT 2 are quite frequent in the region.

The findings of this study highlight the challenges involved in the diagnosis and control of FMD in endemic areas and emphasize the need for optimization of the methods used for serological diagnosis and for serotyping of FMDV outbreaks. There is need for more studies to investigate detailed epidemiology of FMD in wildlife in Uganda.

## Conclusions

African buffalos are important for the maintenance of FMDV within National Parks of Uganda. They play an important epidemiological role in the circulation of FMDV serotypes SAT 1 and SAT 2, and may also harbour serotype SAT 3 and O infections.

## Methods

### Study area

**Figure 4:**
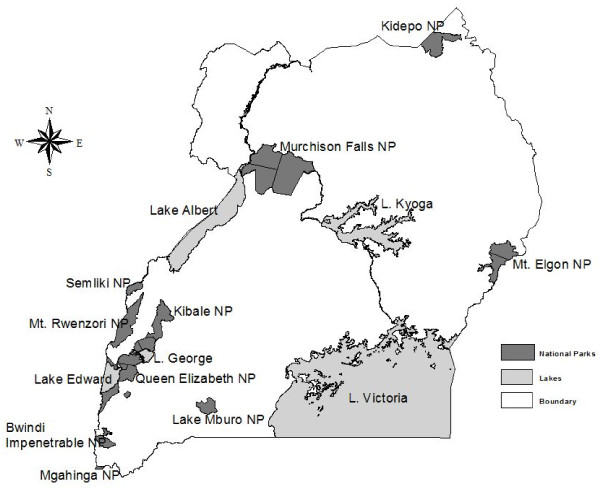
**Map of Uganda showing the location of the National Parks**. NP stands for the National Park.

The present study was kindly approved by Uganda Wildlife Authority (UWA/PMR/RES/50) and wildlife samples were collected from four major National Parks in Uganda, namely; QENP, LMNP, MFNP and KVNP (Figure 4). These National Parks were chosen on the basis of the high chance of livestock-wildlife interactions. Compared to other National Parks in Uganda, they are generally flat or gently sloping and not densely covered by vegetation thereby facilitating the exercise of darting and follow up of the sedated animals. Such National Parks are also home to sizeable buffalo populations with estimates of about 6,807 animals in QENP, 132 in LMNP, 8,200 in MFNP and 400 in KVNP [37]. All the national parks are unfenced and hence provide possibilities for livestock-wildlife interactions.

Due to the large buffalo population and the very high chances of livestock-wildlife interactions, more samples were collected in QENP than in the other parks.

### Sampling

Apart from the impala, chemical capture was used for immobilization of animals of choice [38,39]. The original target of sampling at least 10% of each herd was not possible. Most buffalo herds would disperse and sometimes scatter to inaccessible areas upon darting one or a few of them. At times it would be impossible to locate herds in the National Parks. Animals were darted with a Dan-Inject dart gun. Two cars were used; one for the identifying and darting the animals and the other for tracking the herds, general field support and tracing the darted animal. Buffalo herds were located and animals moving at the edge of the group identified and darted. The anaesthetic combination was 8-10 mg Etorphine (Kyron, South Africa) and 70-90 mg Xylazine (Kyron, South Africa). The sedated animal would be cautiously located and approached, held by the horns and head, blindfolded and the mouth opened and the tongue pulled out for examination for lesions and ensuring continuous respiration before collection of serum and probang samples. After sampling, the sedative was reversed by use of a combination of 14-18 mg Diprenorphine and 60-70 mg Yohimbine (Kyron, South Africa) by intravenous infusion through the ear vein. The age of the buffalos was estimated from the teeth. All buffalos fell within the age group used for rinderpest serosurveillance (1.8-20 years). Non-buffalo species other than the impala were also darted following similar techniques as defined by Kock et al. [39]. Due to significant challenges of chemical capture, impala were instead physically restrained after dazzling them with strong light directed at the eyes at night time, during periods of little or no moonlight [40].

A total of 134 African buffalo samples and 21 impala samples were collected during 16 trips in the years 2007 and 2008 (Table 1). The samples from giraffe (1), hartebeest (7) and waterbuck (5), were jointly obtained through the on-going wildlife health research and monitoring programmes by Uganda Wildlife Authority in 2007. Eighty African buffalo samples and 1 Eland sample had been collected during the rinderpest serosurveillance exercise between the years 2005 and 2006. Probang samples were preserved in 0.04 M phosphate buffered saline (PBS), transported under liquid nitrogen while in the field and stored at -80°C at the laboratory. Serum was separated from blood and stored at -20°C in the laboratory.

### Screening for antibodies to FMDV non-structural proteins

207 buffalo samples were screened for antibodies against non-structural proteins (NSP) of FMDV using the commercial Ceditest FMDV NS^® ^kit (Cedi diagnostics BV, Netherlands) [41]. This test is currently marketed as Priocheck^® ^FMDV NS by Prionics^® ^AG, Switzerland. In addition, samples from impala (n = 21), hartebeest (n = 7), waterbuck (n = 5), eland (n = 1) and giraffe (n = 1) were tested in the same way.

### Serotype-specific Solid Phase Blocking ELISA (SPBE)

137 African buffalo serum samples, of which seven were not tested for antibodies against NSP, were screened (dilution 1:5) for serotype-specific antibodies against FMDV using an in-house SPBE system modified from Have and Holm-Jensen [42] and described in detail by Balinda et al. [43]. The O, A, C and Asia 1 tests in this ELISA system have been used at the National Veterinary Institute, Danish Technical University (Lindholm), for many years; they have been validated for cattle and swine (ISO/IEC 17025) and used for many other ruminants and Camelidae with good results, and they appear to work well on all species (Alexandersen, unpublished results). The SPBE tests for antibodies against the SAT-serotypes were more recent and were still undergoing evaluation. Closely related ELISA tests for the SAT-serotypes have been set up and used under African conditions for detecting antibodies against multiple FMDV serotypes and shown to perform well [43,44].

For each well, optical density (OD) as a percentage of the mean OD of four wells with negative control sera (ODP) was calculated according to the formula: ODP = ((sample OD_450 _- OD_620_)/(mean of (negative control sera OD_450 _- OD_620_)) × 100. Samples were considered positive, if ODP was lower than 50% in the antibody tests for O, SAT 1, SAT 2 and SAT 3, 45% for A and 35% for C and Asia 1.

Based on the serological status and availability of sufficient amounts, 37 positive samples were selected and titrated (up to dilution 1:640) in the relevant serotype specific SPBEs. Titres were expressed as the reciprocal of the highest positive dilution.

Due to limited sample volumes and the smaller number of trips made, serotype specific SPBE studies did not include KVNP.

### FMD Virus isolation and antigen ELISA

The methodology of virus isolation from the OP samples was adopted from the standard procedure described by the World Organisation for Animal Health [45]. Briefly, 50μl of undiluted sample and a 1:10 dilution of the sample were each inoculated into 5 wells of a 96-well microtitre plate with monolayers of primary bovine thyroid (BTY) cells and 100μl of Eagles media with 2% fetal calf serum. A row of wells with negative control sera including buffer was inserted between each sample. The cell cultures were incubated at 37°C and examined for cytopathic effect (CPE) for 2-4 days. Negative cultures were passaged onto new bovine thyroid (BTY) monolayers once. First and second passage cultures with CPE were harvested and serotyped using an in-house antigen ELISA set up at the National Veterinary Institute, Lindholm, Denmark, based on the description by OIE [45]. Briefly, the rabbit and guinea pig hyperimmune sera were the same as used in the in-house SPBE for serotype-specific antibodies against FMDV described above. The samples were tested in duplicate, and for each serotype each plate included two wells with strong positive control sera, two wells with weak positive control sera and two wells with negative control sera, all consisting of cell-culture materials. The tests for serotypes O, A, C and Asia 1 were quality assured (ISO/IEC 17025), while the tests for serotypes SAT 1, SAT 2 and SAT 3 were more recently set up and still undergoing evaluation.

### RNA extraction, RT-PCR and cycle sequencing

Total RNA was extracted from CPE positive cell cultures using the RNeasy-Mini Kit^® ^(Qiagen, Germany) according to the manufacturer's instructions. cDNA was synthesized from the template using Ready-To-Go^® ^You-Prime First-Strand Beads (GE Healthcare Life Sciences, UK) and a four-primer mix of NVT_24 _, A PN 63 (5'- AGACCTGGAAAGACCAGGC-3'), G_15_H, and pdN6 (random hexamers). To generate 15 overlapping PCR fragments for near full length genome sequencing, 15 PCR-tubes were prepared containing: 33.1 μl of water, 5.0 μl 5 × AmpliTaq Gold buffer, 4.0 μl MgCl_2 _(25 mM), 0.4 μl dNTPs (2.5 mM each), 2.5 Units of Amplitaq Gold^® ^(Applied Biosystems, UK) and 5.0 μl of template cDNA. To each of these tubes, 1.0 μl of respective fragment-specific forward and reverse primers, each at a concentration of 25 pmol/μl was added to make a total volume of 50 μl.

The primers used for the VP1 coding region are shown in Table 3. The PCR (Perkin Elmer PE 9700) was set and ran at 95°C for 5 minutes to activate Amplitaq enzyme followed by five cycles (95°C for 15 seconds, 55°C for 30 seconds with less by 1 second in each subsequent cycle and then 72°C for 1 minute and 20 seconds), 40 cycles (95°C for 15 seconds, 50°C for 30 seconds, and 72°C for 1 minute and 20 seconds-adding 1 second per cycle) and lastly at 72°C for 7 minutes and kept at 4°C. To confirm the presence or absence of PCR products, gel electrophoresis was undertaken using 1.2% agarose containing 0.005% ethidium bromide. Amplicons were extracted from the gel using the Qiaquick® (Qiagen, Germany) gel extraction kit and sent to AGOWA (Germany) for cycle sequencing.

**Table 3:** List of primers used for RT-PCR. For each fragment, forward and reverse primers were used

Sample ID	**Forward Primers (**5' to 3')	**Reverse primers (**5' to 3')
**BUF 10**	CAGTACTCCGGCAGCCTG	GGTGTTGTAATTGCACTCTCC
	CAGTGGTGTTCTCGCACAAC	GCCATDGGMGGGATGAACCC
**BUF 6**	GACCGTATTCTCACCACGAG	AAGTTGGACCTGACGTCGG
**BUF 17**	CAAAXAGGGAATTTTXCCCGTXGC	GACGACXGGXTTGTCGCC
	CTGGTXGGCGCAATCCTXCGT	CGGTTRAAGTCGGGWCCGTG

The sequences obtained from samples BUF 10, BUF 6 and BUF 17 were subsequently named SAT 2/UGA/1/07, SAT 2/UGA/2/07 and SAT 1/UGA/1/07, respectively. These samples were all collected on the same day (17/1/07) in Queen Elizabeth National Park, but BUF 17 was from a different herd.

### Sequence analysis

A phylogenetic tree of the virus sequences was inferred using the Neighbor-Joining method [46]. The bootstrap consensus tree inferred from 1000 replicates is taken to represent the evolutionary history of the taxa analyzed [47]. The evolutionary distances were computed using the Kimura 2-parameter method [48] and are in the units of the number of base substitutions per site. The sequences studied were all from the VP1 coding region of the current FMDV isolates and the reference topotypes (Table 4). All positions containing gaps and missing data were eliminated from the dataset. There were a total of 660 nucleotides in the final dataset. Phylogenetic analyses were conducted in MEGA 4 [49,50]. In order to deduce the amino acid sequences, the East African SAT 2 prototype sequences together with the Ugandan buffalo sequences (this study) and those from cattle during 2004 [26] corresponding to the C-terminal part of the VP1, were aligned and translated in MEGA 4 and exported to the Bioedit sequence alignment editor [51] to identify the positions of differences and similarities.

**Table 4:** Summary of the Viruses used in this study

Serotype	Host Animal	Virus strain	**GenBank accession no**.	Country
**SAT 1**	Buffalo	SAT1/UGA/1/07*	HM067706	Uganda
	-	SAT1/T155/71*	N/A	Tanzania
	-	SAT1/ZIM/23/2003	N/A	Zimbabwe
	-	SAT1/RV/11/37	AY593839	Unknown
	-	SAT1/RHO/5/66	AY593846	Rhodesia
	-	SAT1/BEC/1/48	AY593838	Botswana
	-	SAT1/BOT/1/68	AY593845	Botswana
	Buffalo	SAT1/UGABUFF/21/70	N/A	Uganda
	-	SAT1/NIG/11/75	AF431711	Nigeria
	-	SAT1/ISR/4/62	AY593844	Israel
	-	SAT1/SUD/3/76	AY441996	Sudan
	-	SAT1/UGA/13/74	AY442010	Uganda
	-	SAT1/UGA/1/97*	AY442012	Uganda
	Cattle	SAT1/ETH/3/2007	FJ798154	Ethiopia
**SAT 2**	Cattle	SAT2/UGA/01/2004*	GU323171	Uganda
	Cattle	SAT2/UGA/05/2004*	GU323174	Uganda
	Cattle	SAT2/UGA/12/2004*	GU323179	Uganda
	Buffalo	SAT2/UGA/1/2007*	HM067705	Uganda
	Buffalo	SAT2/UGA/2/2007*	HM067704	Uganda
	-	SAT2/SA/106/59	AY593848	Unknown
	-	SAT2/ZIM/14/2002	N/A	Zimbabwe
	Cattle	SAT2/ZIM/7/83*	AF136607	Zimbabwe
	-	SAT2/ZIM/5/81	EF134951	Zimbabwe
	-	SAT2/RHO/1/48	AY593847	Rhodesia
	Buffalo	SAT2/BOT/P3/98	AF367124	Botswana
	Cattle	SAT2/KEN/1/84	AY344505	Kenya
	Cattle	SAT2/ETH/1/90	AY343935	Ethiopia
	Cattle	SAT2/NIG/2/75	AF367139	Nigeria
	Cattle	SAT2/GHA/2/90	AF479415	Ghana
	Cattle	SAT2/GAM/8/79	AF479410	Gambia
	Cattle	SAT2/SAU/6/2000	AF367135	Saudi Arabia
	-	SAT2/CAR/8/2005	N/A	Cameroon
	-	SAT2/ZAI/1/74	DQ009737	DRC
	Cattle	SAT2/RWA/1/00*	AF367134	Rwanda
	Cattle	SAT2/KEN/3/57	AJ251473	Kenya
	Cattle	SAT2/KEN/2/84	AY343941	Kenya
	-	SAT2/ZAI/1/82	AF367100	Zaire
	Cattle	SAT2/UGA/19/98	AY343969	Uganda
	-	SAT2/ANG/4/74	AF479417	Angola
	Cattle	SAT2/UGA/51/75	AY343963	Uganda
	Cattle	SAT2/SUD/6/77	AY343939	Sudan
	Cattle	SAT2/ETH/2/2007	FJ798161	Ethiopia
	Cattle	SAT2/ETH/2/91	AY343938	Ethiopia

*: not WRLFMD reference numbers

-: host animal not indicated

NA: not applicable

## Authors' contributions

CA conceived and designed the study, undertook field work, laboratory studies, data analysis, manuscript preparation, review, corrections and submission. SA, KT, VBM, HRS, ARAO and GJB participated in the supervision of various project activities including field work, laboratory studies, data analysis, manuscript preparation, proof reading and review. FNM participated in field work, laboratory studies, and manuscript proof reading. SNB participated in part of the field work, provision of livestock sequence data and analysis. PN was involved in all the molecular laboratory work. All authors read and approved the final manuscript.
